# View specific generalisation effects in face recognition: Front and yaw comparison views are better than pitch

**DOI:** 10.1371/journal.pone.0209927

**Published:** 2018-12-28

**Authors:** Simone Favelle, Stephen Palmisano

**Affiliations:** School of Psychology, University of Wollongong, Wollongong, New South Wales, Australia; Heriot-Watt University School of Social Sciences, UNITED KINGDOM

## Abstract

It can be difficult to recognise new instances of an unfamiliar face. Recognition errors in this particular situation appear to be viewpoint dependent with error rates increasing with the angular distance between the face views. Studies using front views for comparison have shown that recognising faces rotated in yaw can be difficult and that recognition of faces rotated in pitch is more challenging still. Here we investigate the extent to which viewpoint dependent face recognition depends on the comparison view. Participants were assigned to one of four different comparison view groups: front, ¾ yaw (right), ¾ pitch-up (above) or ¾ pitch-down (below). On each trial, participants matched their particular comparison view to a range of yaw or pitch rotated test views. Results showed that groups with a front or ¾ yaw comparison view had superior overall performance and more successful generalisation to a broader range of both pitch and yaw test views compared to groups with pitch-up or pitch-down comparison views, both of which had a very restricted generalisation range. Regression analyses revealed the importance of image similarity between views for generalisation, with a lesser role for 3D face depth. These findings are consistent with a view interpolation solution to view generalisation of face recognition, with front and ¾ yaw views being most informative.

## Introduction

An ability to successfully recognise new instances and views of an unfamiliar face is critical for most of our interactions involving faces, including common tasks such as face matching, face classification and face identification. While theories of familiar face recognition suggest that accurate identification should occur regardless of viewpoint, for example, see [1], this ability does not appear to generalise to the recognition of unfamiliar faces [2]. Despite a long history of research into viewpoint effects on object recognition (see [3]), this aspect of face perception is less well understood. While there are likely to be some similarities in the mechanisms involved in generalising across views for objects and faces, there also appear to be important differences. For example, it has been shown that recognisability and patterns of view generalisation are markedly different for upright and inverted faces [4]. Such findings suggest that our ability to generalise across views of faces is not simply based on low-level image properties but also utilises the class-based knowledge that we have for upright faces [5].

Much of the past research into face recognition has focused on the front or “full-face” view with good reason (but see applied research on matching with CCTV images [6,7], and work on highly variable face images [8]). Not only does this particular view have social significance, but it also provides clear and unobstructed access to the entire face (i.e., there is no occlusion by any of the facial features). When interacting and conversing with other people, we typically orient our heads to achieve something close to a front view of their face. However, every day we also have to identify faces from a rich variety of other views (such as faces viewed from above or below). This study was therefore designed to investigate visual recognition performance across a broad range of face views. It will examine how the nature and the degree of viewpoint transformation affect our ability to generalise from one face view to another. Mental rotation accounts of viewpoint generalisation propose that a stimulus is mentally rotated to match a stored view (e.g., [9]), and do not differentiate between rotation in one axis and another (for example, mental rotation from a front view through 45° to the right should be similar to mental rotation from a front view through 45° above). View interpolation accounts, on the other hand, involve a view-combination mechanism (e.g., [10]) which suggests that generalisation of face recognition relies on comparisons of image information across different views. We are also interested in whether there are some face views for which this generalisation is easier. Evidence for the existence of such “canonical” views would have multiple benefits. First, it would help us understand the nature of the visual information that is necessary for determining that two images are in fact different views of the same face. More generally it would be highly informative about the nature of the representations underlying face processing (e.g., what exactly is being stored?). In addition, evidence for the view/s with best generalisation of face recognition performance has clear applied value in decisions about information to include in identity documents (e.g., passports).

### Face view generalisation following rotations about the different axes

**Fig 1:**
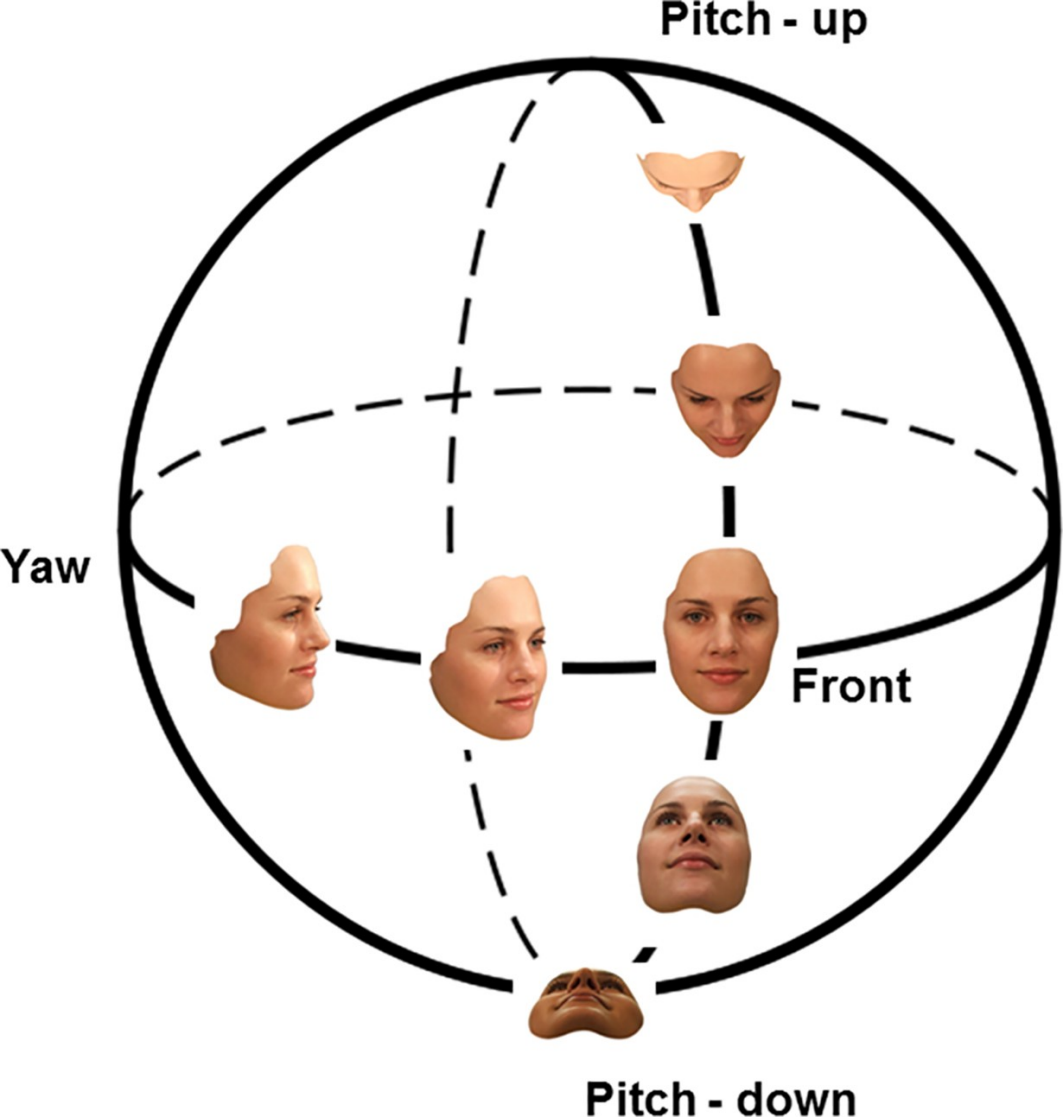
Face views. This viewing sphere shows the images of faces produced by rotating the viewer/camera in yaw or pitch by 0° (the front view), 45° (three-quarter views) or 75°.

Viewpoint dependent effects for unfamiliar face recognition are usually demonstrated for camera or face rotations around a vertical (yaw) axis, which show performance costs in terms of both accuracy/sensitivity and response latency. While these viewpoint dependent costs are known to increase with the angular distance between the probed viewpoints [2], [4], [10], [11], [12], they also depend on the axis of rotation [13]–[15]. One study compared face recognition across camera rotations about the yaw, roll (rotation in the picture plane) and pitch (rotation around the horizontal axis resulting in views from above and below; see Fig 1) with a task in which participants matched sequentially presented face views either to or from a front view [14]. Face recognition was found to be viewpoint dependent for rotations about all axes but was overall best and had the shallowest decline in roll, was poorer and had a steeper decline for rotations in yaw and was poorest and had the steepest decline for rotations in pitch. Further, viewpoint costs were also found to be steeper for faces viewed from above (rotating the camera upwards in the pitch axis) than below (rotating the camera downwards in the pitch axis). These results, along with similar findings from [13], suggest that either: 1) different mechanisms are involved in generalising across views within each axis; or 2) the available information along each axis varies substantially in its utility for face generalisation. These two accounts are not mutually exclusive. While the evidence for different mechanisms is more difficult to obtain, it is obvious that visual face information can vary dramatically following rotations about these three axes (and thus would likely affect the generalisability/consistency of recognition accuracy across views).

The results of Favelle et al. [13] and Favelle et al. [14] are based on matching to or from a front view (the only view common to all three axes). Quite often, however, the views we have to match across do not include a front view. Our previous findings suggest it is more difficult to recognise faces viewed from above and below (pitch) than faces viewed from the left or right (yaw). This pattern of performance is not consistent with a mental rotation account of viewpoint dependence unless rotation of views in the yaw axis is more efficient than rotation in the pitch axis. Further, this pattern of results could be due to the greater image variation across the range of pitch (compared to yaw) rotated views, or to the yaw rotated views containing more useful information for this generalisation task. One way of addressing these questions is to test the degree to which generalisation of face recognition occurs for views rotated about orthogonal axes (e.g., when matching a 45° yaw rotated face view to a pitch rotated face view). First, while there is no a priori reason to expect rotation mechanisms to differ between axes, previous results that show better recognition for yaw than pitch views of faces may be explained by a more efficient rotation mechanism for yaw than pitch axes. If this is the case, then mental rotation between a pitch and yaw view should be as efficient as the mental rotation between a yaw and pitch view. Second, if axis-specific view information or angular distance between face views is important for generalisation of face recognition then the best performance should be seen for within, as opposed to across, axis matching. However, if one view is more “informative” then generalisation should be better from this canonical face view to all other views, including those produced by camera rotations about the orthogonal axis.

Typically, in the study of viewpoint effects, view is changed between study and test along a single axis (e.g., in studies of yaw rotation effects, observers first study front face views and then are subsequently tested with 0°, 45° and 90° yaw rotated face views). Few studies have examined the problem of face recognition when such rotations can occur about orthogonal axes. Wallraven, Schwaninger, Schumacher and Bülthoff [16] examined face recognition for views rotated about the same and orthogonal axes to the learned views. They had participants learn faces at 0° (front) and 60° yaw rotated views and then tested recognition for views rotated in yaw and in pitch (the orthogonal axis). In line with results for novel object recognition [10], the authors found that face recognition performance was superior for yaw, compared to pitch, rotated views at test. Both studies concluded that their results were more compatible with a view interpolation model of object recognition, where view generalisation was based on perceptual similarities in the images, rather than on mental rotation or some other type of transformation. Whether a similar pattern of performance holds for faces learned in pitch and tested in yaw remains to be seen. If axis-specific view information or angular distance plays a key role in generalisation of face recognition, then we would expect to see a complementary pattern of performance, such that after learning a pitch face view, recognition performance will be better when tested with pitch (as opposed to yaw) rotated views. Previously, Bülthoff and Edelman [10] reported that when novel objects are learned in pitch and tested following yaw rotation, there appeared to be little difference in generalisation between axes. However, unlike faces, their visual objects (paperclips and amoeboids) had no intrinsic polarity and so it is unclear whether a similar pattern would also be found when generalising across views of faces.

### Canonical views and the three-quarter view advantage

A canonical view of an object is a view that is most informative or representative of the visual aspects of that object and thus results in superior object naming and recognition compared to other views [17]. In face recognition, the three-quarter view (i.e., ¾ yaw view) has been identified as a likely candidate for a canonical view. It is generally considered to lay approximately half way between a front view and a profile view ([18]; see Fig 1). Since a front view is typically labelled as 0° and a profile view as 90°, a three-quarter view is a left/right yaw rotation of approximately 45° (+/- 15°).

In studies where the learning and test views are identical, a “same-view advantage” has been found for ¾ yaw (compared to front and profile) views (see [12] and [19] for human research, and [20] for related computer vision research; see also [16] for a review). However, ¾ yaw views also appear to be better for generalising to other views (i.e., a “different-view advantage”—see [5], [15], [21], [22]). Hill et al. [4] found that learning a 0° (front) view saw performance decline as a function of the angular distance between study and test views, whereas learning a 45° yaw view saw recognition peak for the opposite 45° yaw view (learning a 90° profile view resulted in poor performance for all unlearned views). Similarly, Van der Linde and Watson (Experiment 2, [15]) found that a 30° yaw rotated view at study generalised better to all other roll and yaw rotated views than any other view (i.e., a ¾ view advantage). Hill et al. [4] suggested that three-quarter views contain information about the contour and projection of features and so provide the best conditions for the extraction of three-dimensional (3D) shape. But while this type of “different-view advantage” may be based on intrinsic properties of the view (i.e., its canonical status), it has been difficult to rule out possible effects based on angular distance between the study and test views. The three-quarter view advantage could also be explained by this particular view often being the closer to the other views being tested [15], [18].

The current study will examine the ability to generalise from four different comparison views: (i) a front face view, (ii) a ¾ view from the right (yaw-right), (iii) a ¾ view from above (pitch-up), and (iv) a ¾ view from below (pitch-down). This will allow us to distinguish between the competing accounts of the three-quarter view advantage. The angular distance between a yaw ¾ view and a front or pitch rotated test view will be the same as that between a pitch ¾ view and a front or yaw rotated test view. Thus, if angular distance is the primary determinant of this three-quarter view advantage [18] then there should be no difference in the pattern of results for yaw and pitch ¾ views. However, if the intrinsic properties of a yaw ¾ view provide critical information then we might expect this particular view to generalise best both to other yaw and to other pitch rotated views.

### Role of image similarity and perceived 3D shape in generalising across views

In addition to the angular distance between the different face views, their image similarity [23], [24], and their perceived 3D shape [4], [25] might also be important for view generalisation of face recognition. View interpolation accounts of generalisation place an emphasis on image comparison to solve the problem of deciding whether two images represent the same identity and would predict that greater image similarity would lead to higher accuracy in this task.

Image similarity may be estimated in a variety of ways–such as by an observer simply rating the similarity of the two images (a high-level subjective measure) or by comparing the number of pixels in the two images that correspond to the face (a lower-level physical measure; see [26] and [27] for studies that have used this measure in examining object recognition). Face images produced by equivalent yaw and pitch rotations clearly differ in terms of the latter physical measure. For example, raw pixel counts show that views rotated more than 45° in pitch provide significantly less visual face information than the equivalent rotations in yaw (see S1 Appendix). While we would anticipate that participant ratings of image similarity would reveal strong differences for similarly extreme pitch views, other aspects of the image (for example, colour or feature occlusion) are also likely to influence ratings of this kind. In the current study, we will examine the role that image similarity plays in view generalisation of face recognition (based on analyses of both similarity ratings and pixel differences between views).

Better perceptions of 3D face shape may also assist in view generalisation of face recognition. Hill et al. [4] have previously proposed that a 45° yaw rotated view provides good conditions for extracting 3D shape, and should therefore lead to more successful view generalisation of face recognition (compared to front and profile views). Stereopsis, which offers additional information about the 3D shape and structure of the face not available in 2D images, has been shown to provide an advantage for generalising across views of faces over flat or synoptic 2D images [28], [29]. A recent eye-tracking study also provides evidence that features such as the nose and cheeks (which have no projection information in a front view) become more salient with stereoscopical viewing [29]. Yaw rotation changes the external contours of an object which is thought to be important for object recognition across rotations in depth [30]. Pitch rotation, on the other hand, results in relatively greater changes to internal facial features such as foreshortening and occlusion/accretion (compared to the changes made to the external contour). However, this internal feature information may also be beneficial for generalising across different face views—since similar internal information in line drawings and shaded objects (but not silhouettes) has been shown to be important for identifying objects rotated in depth [31], [32]. Thus, in this study we will also use a depth estimation task as a measure of the perceived 3D shape information provided by each view.

### The current study

The purpose of this study is twofold. Where previous research predominantly uses a front view as comparison, here we also test ¾ yaw right, ¾ pitch-down, and ¾ pitch-up comparison views. First, we wish to determine whether differential patterns of viewpoint costs across yaw and pitch axes found in previous research (e.g., [13], [14]) are contingent on matching to a front view. While patterns of performance in previous studies do not appear to be consistent with mental rotation accounts of view generalisation, those results may be explained by a more efficient rotation mechanism for yaw than pitch axes. If this is the case then performance matching pitch to yaw views should be similar to matching yaw to pitch views. That is, performance in the ¾ yaw right, ¾ pitch-down, and ¾ pitch-up comparison groups should be similar. Second, we will test whether any of the four different comparison views (front face, ¾ yaw right, ¾ pitch-down, and ¾ pitch-up) have canonical status by examining view generalisation of face recognition both within axis and across orthogonal axes (a canonical view should confer its generalisation advantage to rotations across axes as well as within). In addition to any intrinsic properties of the images, we will examine the extent to which angular distance, image similarity and differences in 3D shape information between face views accounts for successful view generalisation of face recognition in line with view interpolation accounts of generalisation.

Specifically, to address these aims we used a sequential matching task to measure generalisation of face recognition from a particular comparison view to multiple test views. Comparison (or first) view will be examined as a between-subjects group factor. The comparison view presented on each trial will differ for each group (either front face, ¾ yaw right, ¾ pitch-down, or ¾ pitch-up). The test faces will be the same for all groups and consist of different views rotated in yaw from 75° left of 0° through to 75° right of 0° (in 15° increments) and of views rotated in pitch from 75° below 0° through to 75° above 0° (i.e., rotation was manipulated independently within each axis).

## Methods and materials

### Participants and design

A total of 80 undergraduate psychology students from the University of Wollongong served as participants for this experiment and received course credit for participation (see section 2.3 for sample size rationale). Their ages ranged from 18 to 51 years (*M* = 22.4 years, *SD* = 5.4). Note that one participant did not indicate their age, so N = 79 for the descriptive statistics for age. All had normal or corrected-to-normal vision and none were familiar with the face stimuli used in the experiment.

The experiment had a mixed design with one factor (comparison view) manipulated between subjects and two within subjects factors (test view axis and test view angle). The between subjects factor of comparison view had four levels (each n = 20, see power analysis below). The two within subjects factors were test view axis with four levels (yaw-left, yaw-right, pitch-up and pitch-down) and test view angle with six levels (0°, 15°, 30°, 45°, 60° and 75°). These two factors were combined factorially. Since 0° was common to all axes, this resulted in 21 different test view combinations. The dependent variables were recognition sensitivity (*d’*) and reaction time (RT) measured in milliseconds.

We have previously observed large effect sizes for the main effects and interactions of a similar study of test view axis and angle using front comparison views [14]. A power analysis using the G*Power 3.1.9.2 computer program [33] with the statistical power level set to (1 − β) = .80 and the α-level set to α = .05 indicated that we needed a sample size of 20 to detect an effect size of ηp^2^ = .2 or larger in the context of a 4 (test axis) x 5 (test angle), within-subjects ANOVA. See Table 1 for details of the participant demographics for each group. Ethical approval for this experiment was obtained from the University of Wollongong Human Research Ethics Committee, in accordance with Australian National guidelines.

**Table 1:** Participant demographics by group (each n = 20).

	Front group	¾ yaw group	¾ pitch-up group	¾ pitch-down group
**Comparison view**	Front 0°	45° right yaw	45° pitch-up	45° pitch-down
**Sex**	15 female^a^	15 female	15 female	14 female
**Age in years (SD)**	Range = 18–51. *M* = 22.1 (7.9)	Range = 19–46. *M* = 23.7 (6.0)	Range = 18–27. *M* = 21.8 (2.2)^b^	Range = 18–36. *M* = 22.1 (4.1)

^a^ One participant did not indicate their sex.

^b^ One participant did not indicate their age, so n = 19 for the descriptive statistics for age in this group.

### Stimuli

**Fig 2:**
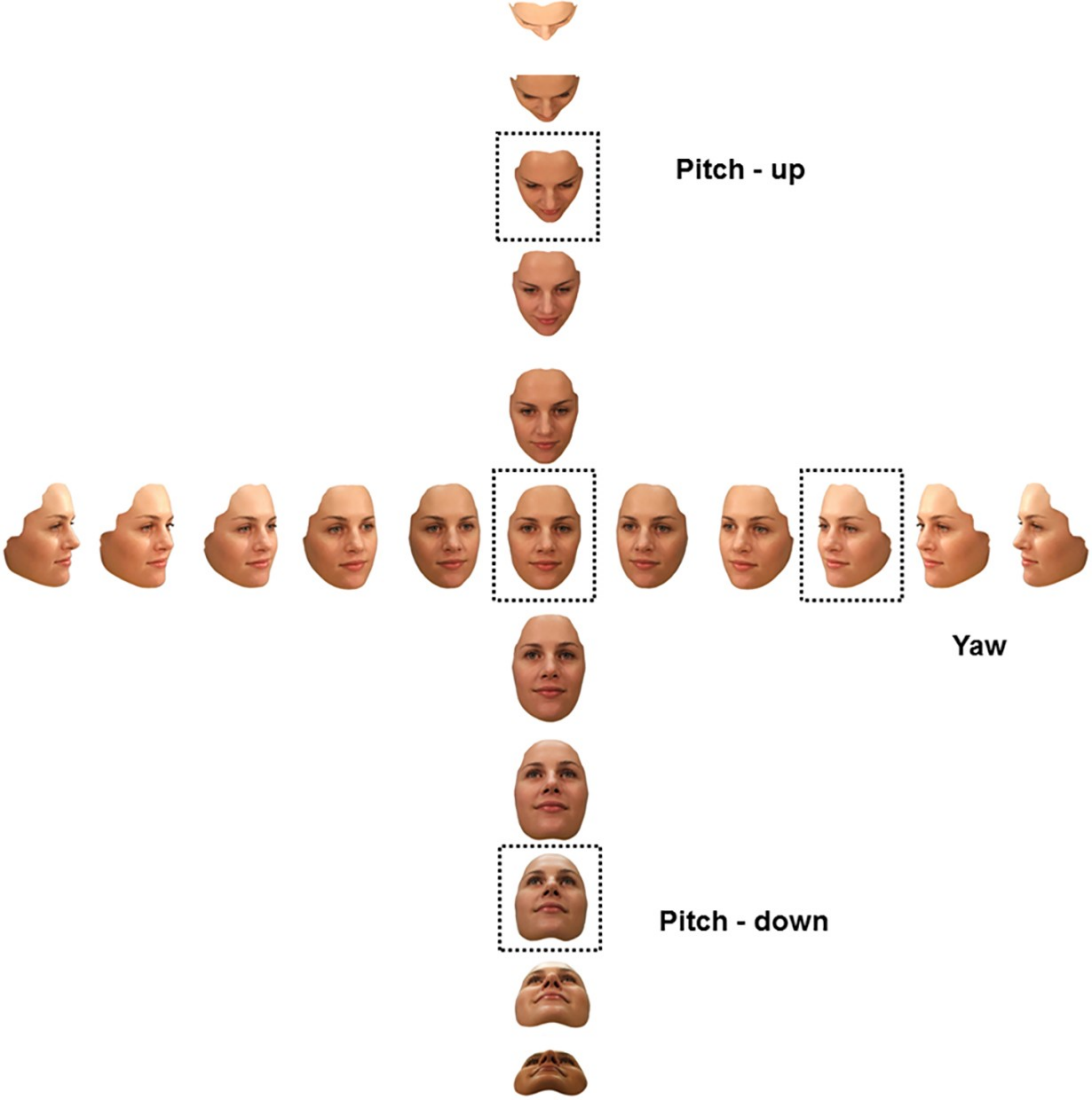
Example face views. This figure shows images of one identity from each of the 21 viewpoints used as test views for all participants. The centre image is a front view and nominally the 0° view. Views along each axis are in 15° increments from 0° (15°, 30°, 45°, 60° and 75°). The views identified by dotted lines are the comparison views for our four different groups (front face, ¾ yaw right, ¾ pitch-down, and ¾ pitch-up).

Stimuli were images of 9 Caucasian female faces taken from a database of high-quality digital face images (see [13], [14]). Faces portrayed a neutral expression, and any distinctive features as well as hair were removed. Lighting (from above and ambient) was held constant across all viewpoints. That is, the camera was rotated around a stationary head to capture the face images. In addition to the front face view (0°), each face was captured from 10 different viewpoints rotated 15°, 30°, 45°, 60° and 75° either side of 0° in pitch and to the right in the yaw axis (left yaw views were created as mirror images of the right yaw views). In total there were 21 different viewpoints generated for each face: full-face 0°, left and right yaw viewpoints of 15°– 75°, pitch-up and pitch-down viewpoints of 15°– 75° (See Fig 2). The individual whose face is portrayed in the stimuli used in the figures in this manuscript has given written informed consent (as outlined in PLOS consent form) to publish these images.

All images were viewed in the centre of the computer screen against a white background. The visual area subtended by 0° face images was 14.7° x 19.2°. For the yaw rotated viewpoints the height of the face image remained constant, however face width increased as the viewpoint was rotated further away from 0°. Face width remained constant for pitch camera rotations, however face height decreased as the viewpoint was rotated further away from 0° (for both pitch-up and pitch-down conditions). The smallest image for the pitch-up camera condition was at a viewpoint of 75°, which produced a visual angle of 14.7° x 9.2°. The rectangular patterned mask used in the experiment subtended a visual area of 18° x 22° and was composed of various elements taken from the stimuli used in the task.

Full colour images were presented on a 48 cm flat-screen monitor with a resolution of 1024 x 768 pixels. Trials were run on a Macintosh G4 computer and RSVP experimental software (Version 4.0.5; www.tarrlab.org) guided the trial sequence.

### Procedure

The experiment took place in a dimly lit room, participants were tested individually. Written consent for participation was obtained and participants were randomly assigned to one of the four comparison view groups (front, ¾ yaw, ¾ pitch-up, ¾ pitch-down). The experiment began with a set of written instructions and a practice session of 14 trials using different faces shown under the same conditions. After the practice trials, participants had the opportunity to ask any further questions before continuing on to the experiment. The experiment consisted of 378 trials (21 viewpoints x 9 identities each for same and different trials). In half of the trials the two faces presented were the same, regardless of viewpoint (same trials). In the remainder the two faces were different; the different face was randomly selected from the images of the remaining 8 face models (different trials). Trial type was presented in random order. Participants were given 6 self-timed rest periods spaced equally throughout the experiment. The experiment lasted approximately 30 minutes.

**Fig 3:**
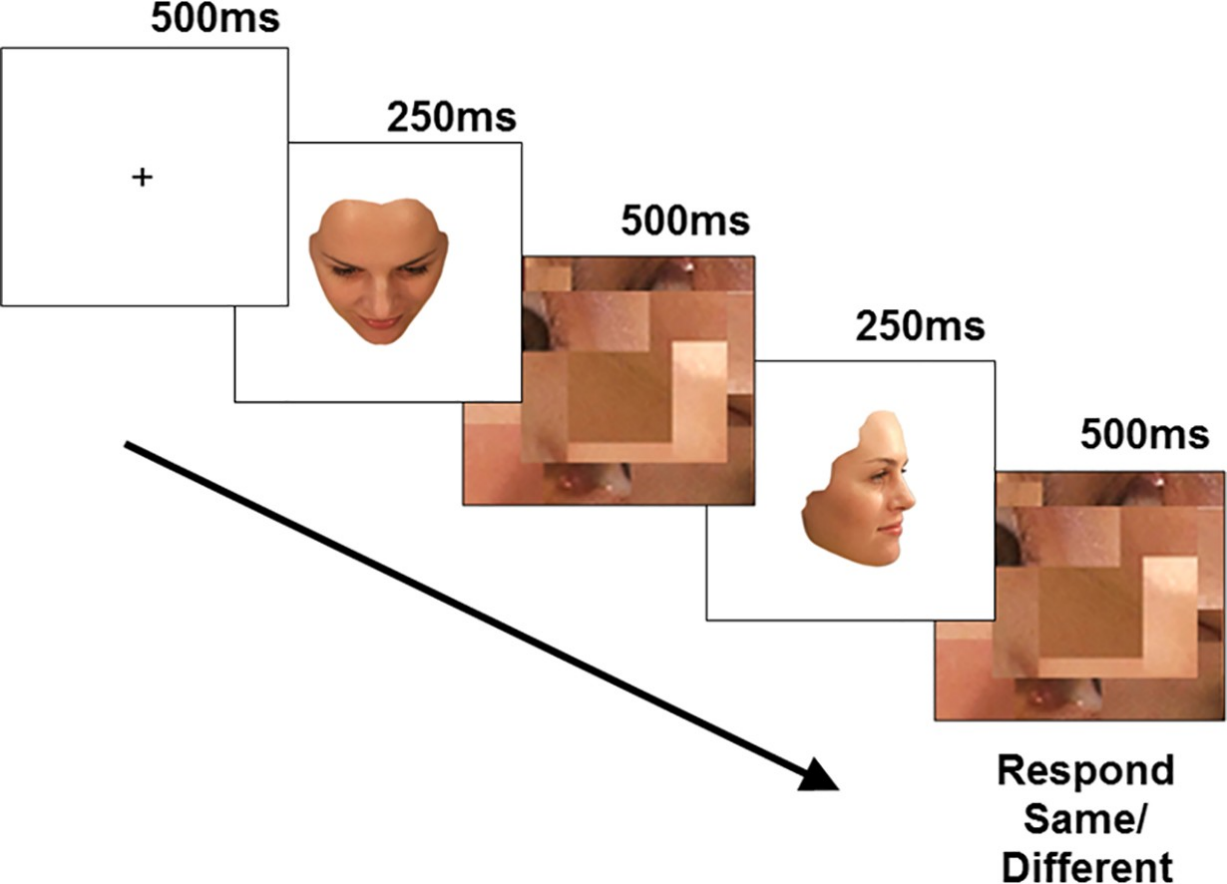
Trial sequence and timing. Face 1 was always the comparison view (in this case the participant was in the ¾ pitch-up group). Face 2 could be any one of the 21 views rotated in yaw or pitch. The identity of Face 2 could either be the same as Face 1 (as it is here) or different (i.e., it could be an image of one of the 8 other identities). Masks were presented in between and directly after these two face stimuli.

Each trial began with a fixation cross displayed for 500 ms followed by the presentation of face 1 for 250 ms. Face 1 was always shown at the same view (front, ¾ yaw, ¾ pitch-up, or ¾ pitch-down depending on the group to which the participant was assigned). Following the presentation of Face 1, a mask was presented immediately after for 500 ms to control stimulus exposure. Face 2 (one of 21 views rotated in either pitch or yaw) was then presented for 250 ms, followed by a second presentation of the mask for 500 ms. Following the second mask the screen remained blank for 2 s or until a response was made by the participant (see Fig 3 for an illustration of the trial sequence). If a response was not made within this time, the trial ended (i.e. ‘timed-out’). The interval between trials was 1 s. Participants were required to respond by pressing ‘same’ and ‘different’ keys (clearly labelled) on a keyboard depending on whether they judged face 1 and face 2 to be the same or different. Less than 0.6% of the total experimental trials (7560) in each group timed out. Trials that timed out were not included in any analyses.

## Results

Analyses were conducted on matching sensitivity (*d’*) and RT for correct trials. Participants’ “same/different” responses were converted into hits and false alarm rates, where a hit was a correct “same” response to a test face, and a false alarm was an incorrect “same” response to a “different” test face. Hits and false alarm rates were converted into *z*-scores and used to calculate *d’* using the formula *d’* = z(Hits)–z(False Alarms) (see [34]). The data were first subjected to two separate omnibus mixed design 4 (comparison stimulus group: front, ¾ yaw, ¾ pitch-up and ¾ pitch-down) x 4 (test view axis: yaw left, yaw right, pitch-up and pitch-down) x 5 (test view angle: 15°, 30°, 45°, 60° and 75°) ANOVAs. Note that zero was not included in this analysis because it was common to all axes. Unless otherwise stated, the statistical analyses had an alpha level of .05 and post hoc comparisons were Bonferroni adjusted. Where the assumption of sphericity was violated, a Greenhouse-Geisser correction was applied to the degrees of freedom.

Next we investigated the possible relationships between matching sensitivity and three potential predictors (the angular distance, image similarity and perceived depth differences between face views) via a multiple regression analysis.

### Analysis of sensitivity (d’) data: Omnibus analysis

**Fig 4:**
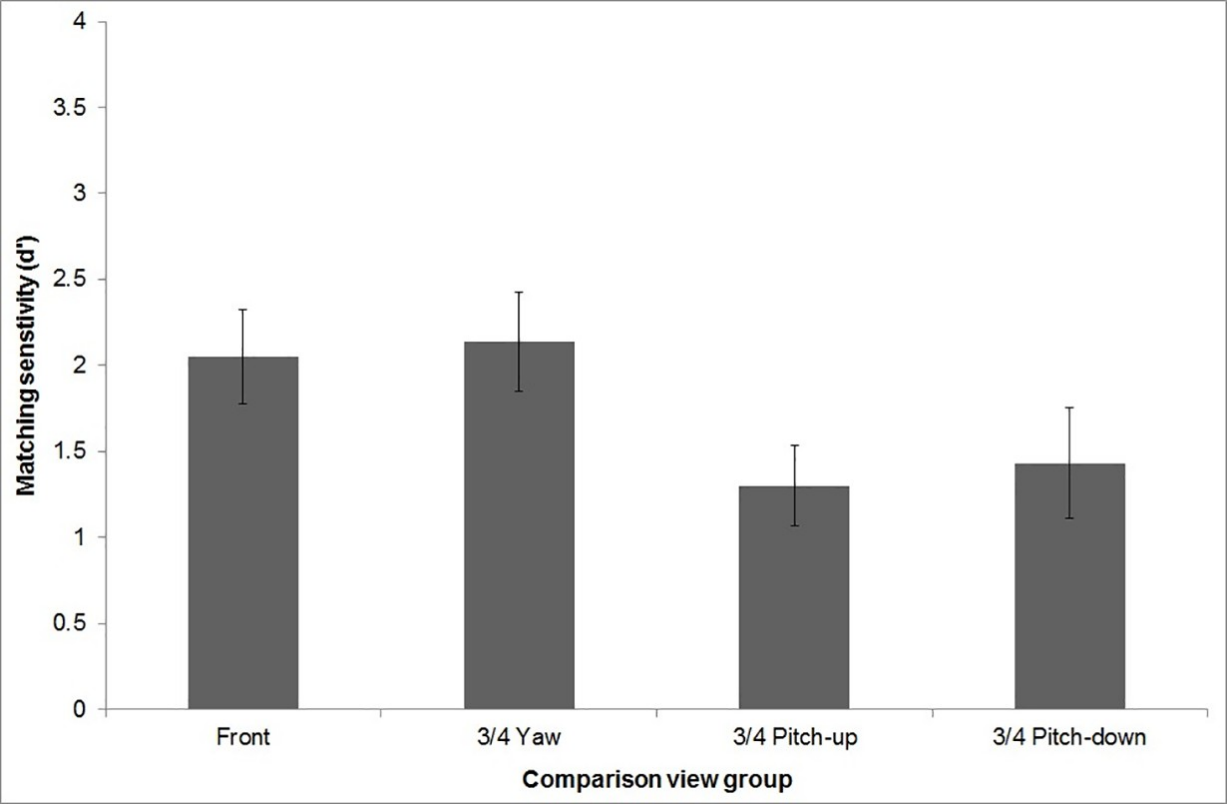
Group sensitivity (d’) data. Overall matching performance is shown for each of the comparison view groups. Error bars represent +/-1 SEM.

Fig 4 displays the overall sensitivity of each group. As can be seen, overall sensitivity was higher when the comparison stimuli were front or 3/4 yaw faces compared to 3/4 pitch-up or 3/4 pitch-down, which is inconsistent with a mental rotation account. These observations were confirmed by the omnibus mixed design ANOVA which showed a significant main effect of group, *F*(3, 76) = 10.40, *p* < .001, η_p_^2^ = .29. Post hoc pairwise comparisons showed that overall sensitivity was higher when the comparison stimuli were front or ¾ yaw faces compared to ¾ pitch-up or ¾ pitch-down faces (all *p* < .01). On average, sensitivity was not significantly different for front face compared to the ¾ yaw comparison stimuli nor was it significantly different for the ¾ pitch-up and ¾ pitch-down comparison stimuli (both *p* = 1.0).

The omnibus mixed design ANOVA also revealed performance differences across the test views in line with the pattern of results found in our previous research [13], [14]. There was a significant main effect of test view axis, *F*(3, 228) = 60.92, *p* < .001, η_p_^2^ = .45in which overall sensitivity was greatest for the yaw rotated test stimuli, with no significant difference in performance for left and right yaw rotated test stimuli (*M*_*YL*_ = 2.02, *SD*_*YL*_ = 1.18, *M*_*YR*_ = 2.00, *SD*_*YR*_ = 1.23; *p* = 1.0). Overall sensitivity for yaw rotated test stimuli was significantly higher than that for either pitch-up or pitch-down rotated test stimuli (both *p* < .001), and overall sensitivity for the pitch-up test stimuli (*M*_*PU*_ = 1.32, *SD*_*PU*_ = 1.27) was lower than that for the pitch-down test stimuli (*M*_*PD*_ = 1.58, *SD*_*PD*_ = 1.09; *p* < .001).

Overall performance was viewpoint dependent, shown by a significant main effect of test view angle, *F*(4, 304) = 59.22, *p* < .001, η_p_^2^ = .44. A linear contrast revealed a significant linear decrease in sensitivity as the test view angle increased from 15° to 75°, *F*(1, 76) = 149.2, *p* < .001, η_p_^2^ = .66.

There were significant two-way interactions between group and test view axis, *F*(9, 228) = 20.18, *p* < .001, η_p_^2^ = .44, between group and test view angle, *F*(12, 304) = 10.40, *p* < .001, η_p_^2^ = .29 and between test view axis and test view angle, *F*(12, 912) = 10.76, *p* < .001, η_p_^2^ = .12. These interactions were, however, all qualified by a significant three-way interaction, *F*(36, 912) = 3.88, *p* < .001, η_p_^2^ = .13. This three-way interaction was examined with a series of four separate fully-within subject ANOVAs.

### Analysis of sensitivity (d’) data: Group analyses

**Fig 5:**
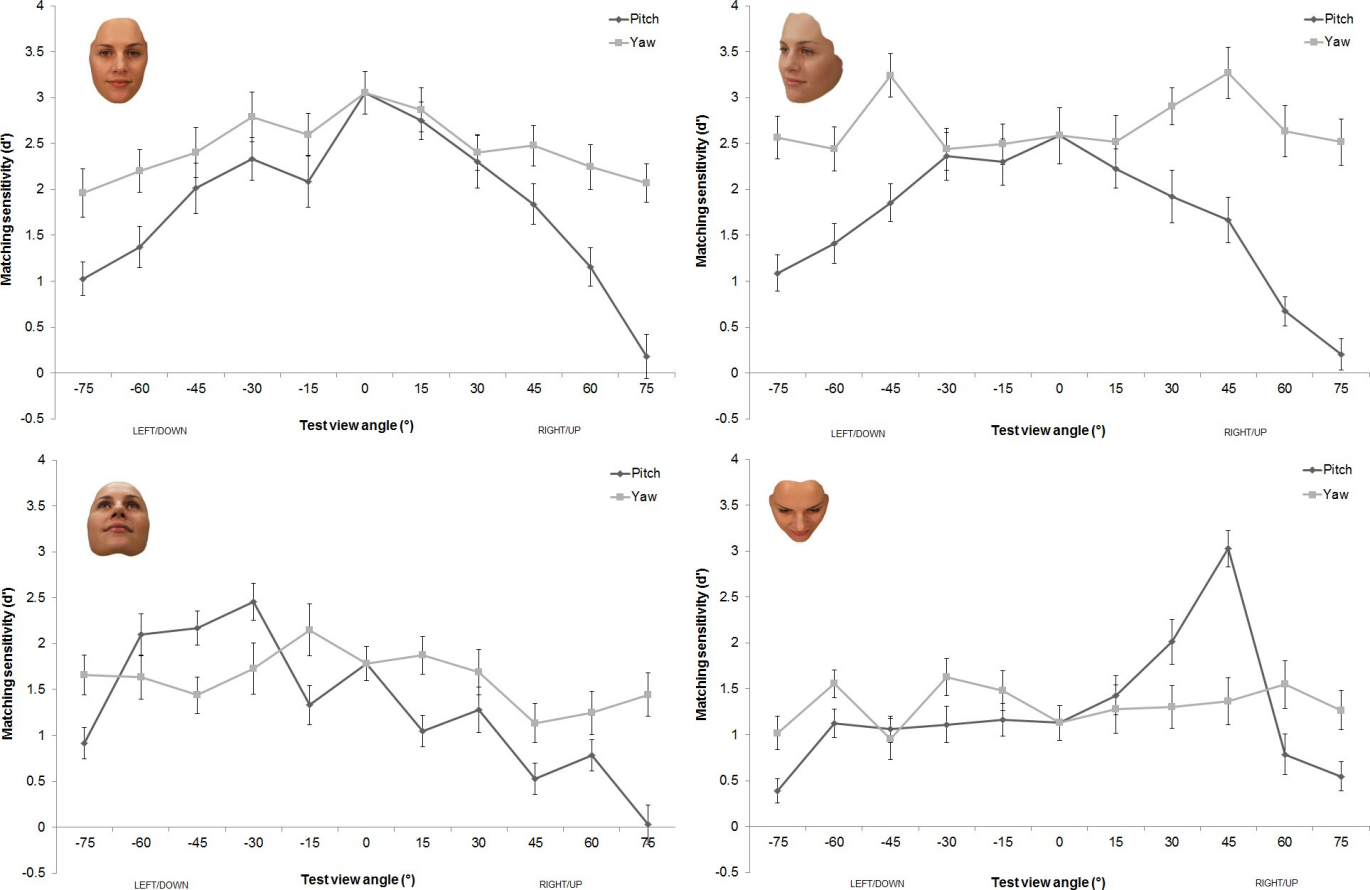
Sensitivity (d’) data. Matching performance as a function of test view axis and test view angle is shown for each of the comparison view groups: front view (top left panel), ¾ yaw view (top right panel), ¾ pitch-down view (bottom left panel), and ¾ pitch-up view (bottom right panel). Error bars represent +/-1 SEM.

We conducted separate 4 (test view axis: yaw left, yaw right, pitch-up and pitch-down) x 5 (test view angle: 15°, 30°, 45°, 60° and 75°) ANOVAs on the d’ data for each of the comparison view groups (front, ¾ yaw , ¾ pitch-down and ¾ pitch-up—see Fig 5 for the four different sets of data).

#### Front view comparison group

For this front view comparison group, the 4 x 5 repeated measures ANOVA showed a significant main effect of test view axis on matching sensitivity, *F*(3, 57) = 12.43, *p* < .001, η_p_^2^ = .40. Post hoc comparisons show greater overall sensitivity for the yaw, compared to the pitch, rotated test views (all *p* < .02). On average, sensitivities to the left and right yaw rotated test views were not significantly different (*M*_*YL*_ = 2.39, *SD*_*YL*_ = 1.15, *M*_*YR*_ = 2.41, *SD*_*YR*_ = 1.01; *p* = 1.0). Sensitivities to the pitch-up and pitch-down rotated test views were also not significantly different on average (*M*_*PU*_ = 1.65, *SD*_*PU*_ = 1.37, *M*_*PD*_ = 1.77, *SD*_*PD*_ = 1.16; *p* = 1.0). There was also a significant main effect of test view angle, *F*(4, 76) = 26.98, *p* < .001, η_p_^2^ = .59. A significant linear contrast revealed a linear decrease in sensitivity as test view angle increased from 15° to 75°, *F*(1, 19) = 100.77, *p* < .001, η_p_^2^ = .84.

These main effects were qualified by a significant test view axis by test view angle interaction, *F*(12, 228) = 3.59, *p* < .001, η_p_^2^ = .16, which replicated previous research [13], [14]. As can be seen in Fig 5, for this front view comparison group, matching performance was best for test views at or around 0° (the mean d’ = 3.0 found for this test view here appears to represent a “same-view advantage”). For each of the four different test view axis conditions, matching sensitivity declined as the test view angle increased–with the greatest viewpoint dependent decline in performance being observed for the pitch-up test views. Linear contrast analyses showed that the effects of test view angle were: 1) similar for yaw-left, yaw-right and pitch-down test views (all *p* > .1); 2) greater for the pitch-up axis test views (compared to the pitch-down axis (*p* = .003) and both yaw axes views (all *p* < .001)).

#### Three-quarter yaw view comparison group

For this ¾ yaw comparison group there was also a significant main effect of test view axis on matching sensitivity, *F*(3, 57) = 84.62, *p* < .001, η_p_^2^ = .82 which showed the same pattern as the omnibus ANOVA (see Fig 5, top right panel). There was no significant difference in matching sensitivity between the left and right yaw test views (*M*_*YL*_ = 2.64, *SD*_*YL*_ = 1.06, *M*_*YR*_ = 2.77, *SD*_*YR*_ = 1.17; *p* = 1.0). However sensitivity for yaw test views was significantly higher than that for either pitch-up or pitch-down test views (both *p* < .001). Matching sensitivity for pitch-up test views (*M*_*PU*_ = 1.34, *SD*_*PU*_ = 1.24) was lower than for pitch-down test views (*M*_*PD*_ = 1.80, *SD*_*PD*_ = 1.11; *p* < .001). The main effect of test view angle was significant, *F*(4, 76) = 20.24, *p* < .001, η_p_^2^ = .52 with a significant linear contrast showing an overall linear decrease in sensitivity as the test view angle increased from 15° to 75°, *F*(1, 19) = 41.35, *p* < .001, η_p_^2^ = .69.

These main effects were qualified by a significant test view axis by test view angle interaction, *F*(12, 228) = 5.14, *p* < .001, η_p_^2^ = .21. For this ¾ yaw comparison group, performance was best for both the 45° yaw-left and 45° yaw-right test views (the mean d’ = 3.24 and 3.27, respectively, found for these conditions appear to represent a same-view advantage, as well as an advantage for its mirror image). Matching sensitivity for the yaw test views appeared to decline as the angular distance increased from either of these two optimal test views (see Fig 5, top right). By contrast matching sensitivity for the pitch test views appeared to decline as the test view angle increased from 15° to 75°. Again performance for the pitch-up test views appeared to be the most affected by altering the test view angle. Our linear contrast analyses showed: 1) no significant difference in d’ as a function of test view angle for yaw-left or yaw-right test views (*p* = .65); 2) significant differences between the pitch and yaw test views (all *p* < .001) and 3) significant differences between the pitch-up and pitch-down test views (*p* < .001).

#### Three quarter pitch-down view comparison group

While there was also a significant main effect of test view axis for the ¾ pitch-down comparison group (see Fig 5, bottom left panel), *F*(3, 57) = 36.79, *p* < .001, η_p_^2^ = .66, this appeared to be markedly different to the effects found for the front and ¾ yaw comparison groups. Overall sensitivity for matching pitch-down (*M*_*PD*_ = 1.79, *SD*_*PD*_ = 1.06), yaw-left (*M*_*YL*_ = 1.72, *SD*_*YL*_ = 1.10) and yaw-right (*M*_*YR*_ = 1.48, *SD*_*YR*_ = 1.04) test views was not significantly different (all *p* > .09). Overall sensitivity to pitch-up test views (*M*_*PU*_ = 0.73, *SD*_*PU*_ = 0.97) was, however, significantly lower to that for the other axis test views (all *p* < .001). The main effect of test view angle, *F*(4, 76) = 11.63, *p* < .001, η_p_^2^ = .38, with a significant linear contrast showing an overall linear decrease in sensitivity as test view angle increased from 15° to 75°, *F*(1, 19) = 19.96, *p* < .001, η_p_^2^ = .51.

There was also a significant test view axis by test view angle interaction, *F*(12, 228) = 4.78, *p* < .001, η_p_^2^ = .20. For this ¾ pitch-down comparison group, the test view angle appeared to have little effect on matching sensitivity for the yaw test views–both the yaw-left and yaw-right functions appeared to be relatively flat. There was clearer evidence of viewpoint dependence in the matching performance for the pitch test views. Specifically, matching performance was best for pitch-down test views from 30° to 60° (mean d’s of 2.1–2.5 were found near the 45° pitch-down comparison view), while performance for pitch-up test views decreased steadily with increasing test view angles—reaching chance (i.e., d’ = 0) for the largest (75°) pitch-up view angle. The linear contrasts for the four different test view axis conditions were not significantly different to each other (all *p* > .09).

#### Three-quarter pitch-up view comparison group

The main effect of test view axis for the ¾ pitch-up comparison group, *F*(3, 57) = 11.20, *p* < .001, η_p_^2^ = .37 showed a pattern analogous to the pitch-down group (as can be seen by comparing Fig 5‘s bottom left and right panels). Overall there was no significant difference in sensitivity for matching with pitch-up (*M*_*PU*_ = 1.56, *SD*_*PU*_ = 1.28), yaw-left (*M*_*YL*_ = 1.33, *SD*_*YL*_ = 0.91) and yaw-right (*M*_*YR*_ = 1.35, *SD*_*YR*_ = 1.08) tests views (all *p* > .27). Overall sensitivity for pitch-down test views (*M*_*PD*_ = 0.97, *SD*_*PD*_ = 0.78) was poorer than for the views along the other three axes (all *p* < .02). There was also a main effect of test view angle, *F*(4, 76) = 10.72, *p* < .001, η_p_^2^ = .36, with a significant linear contrast showing an overall linear decrease in sensitivity as angle of test view increased from 15° to 75°, *F*(1, 19) = 14.24, *p* = .001, η_p_^2^ = .43.

There was also a significant test view axis by test view angle interaction, *F*(12, 228) = 9.84, *p* < .001, η_p_^2^ = .34. For this ¾ pitch-up view comparison group, the best matching sensitivity was found for the 45° pitch-up test view (the mean d’ = 3.0 found for this test view here appears to represent a “same-view advantage”), with a steep declines seen in performance as the pitch test view angle either increased or decreased. Again test view angle appeared to have little effect on matching sensitivity along the yaw test view axis–both the yaw-left and yaw-right functions appear relatively flat. Linear contrast analyses showed no significant difference in the linear functions for rotation for yaw-left, yaw-right and pitch down test views (all *p* ≥ .05). The linear functions for test views along the pitch-up axis were steeper than those for both yaw and pitch-down test views (all *p* < .03). However, the performance function for test view rotation was not linear and appeared quadratic, with a peak at the ¾ pitch-up view.

### Analysis of RT data

**Fig 6:**
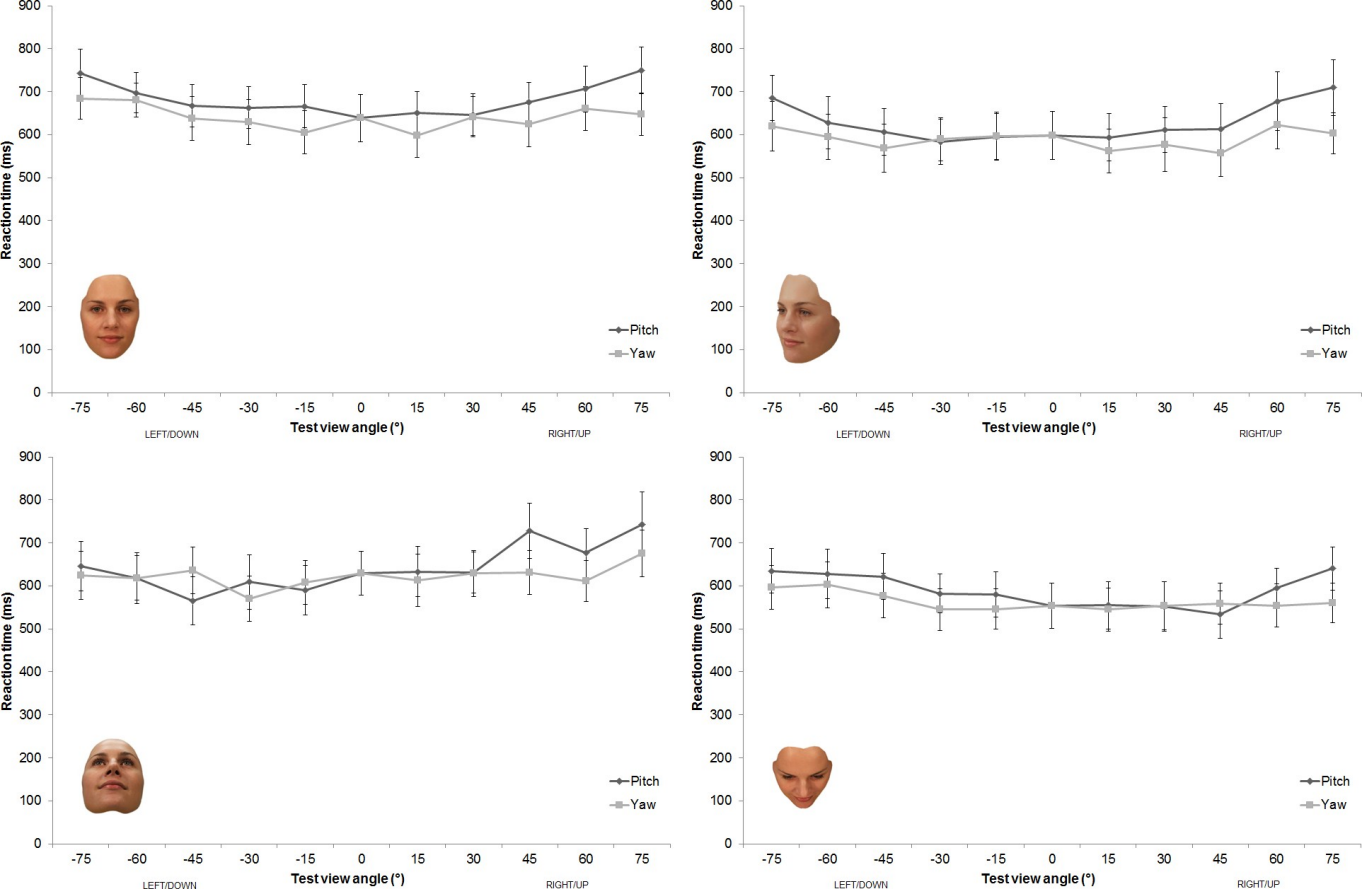
Reaction time data. Reaction time (ms) on the matching task as a function of test view axis and test view angle of rotation for the group comparing: front view faces (top left panel), ¾ yaw view faces (top right panel), ¾ pitch-down view faces (bottom left panel), and ¾ pitch-up view faces (bottom right panel). Error bars represent +/-1 SEM.

Fig 6 displays the reaction time (RT) for each of the four groups as a function of test view axis and test view angle of rotation. As can be seen, the speed of responses in the matching task was similar across groups with RT generally found to be faster for yaw test views compared to pitch. This pattern was confirmed with an omnibus 4 x 4 x 5 mixed design ANOVA showing no effect of group (i.e., comparison view), *F*(3, 76) = .53, *p* = .67, η_p_^2^ = .02, no interaction between group and test view angle, *F*(12, 304) = .87, *p* = .58, η_p_^2^ = .03, and no 3-way interaction between group, test view angle and test view axis, *F*(36, 912) = .99, *p* = .49, η_p_^2^ = .04.

However, both the main effect of test view axis and the interaction between group and test view axis were found to reach significance, *F*(9, 228) = .6.22, *p* < .001, η_p_^2^ = .20 and *F*(3, 228) = 24.07, *p* < .001, η_p_^2^ = .24 respectively. Post hoc comparisons showed that in the ¾ yaw and the front view comparison groups, RT was faster for the yaw (compared to the pitch) test views. However, in the ¾ pitch-up comparison group, RT was similar for the pitch-up and yaw test views and these RTs were all faster than those for the pitch-down test views. The complementary pattern was seen in the ¾ pitch-down comparison group, where RT was similar for the ¾ pitch-down and yaw test views and these RTs were all faster than for the pitch-up test views.

The main effect of test view angle and the interaction between test view angle and test view axis were also found to reach significance, *F*(4, 304) = 26.51, *p* < .001, η_p_^2^ = .26 and *F*(12, 912) = 1.94, *p* = .04, η_p_^2^ = .03 respectively. In general viewpoint dependent costs to reaction time were greater for the pitch (compared to the yaw) test views (see Fig 6). Overall, there was a significant linear increase in RT as test view rotation increased from 15° to 75°, *F*(1, 19) = 66.43, *p* < .001, η_p_^2^ = .47. Post hoc comparisons showed that RTs for yaw axis test views (*M*_*YL*_ = 606.4, *SD*_*YL*_ = 218.9, *M*_*YR*_ = 601.4, *SD*_*YR*_ = 222.9) were faster than those for the pitch-up and pitch-down test views (*M*_*PU*_ = 646.3, *SD*_*PU*_ = 234.2, *M*_*PD*_ = 630.5, *SD*_*PD*_ = 228.3) (all p < .002).

### Relationships between matching performance, angular distance, image similarity and perceived depth

We next conducted a multiple regression analysis to investigate the hypothesis that matching performance (d’) would be a function of the following four variables: (i) the angular distance between the test and comparison views, (ii) the difference in image pixels, (iii) their rated image similarity; and (iv) the difference in the perceived 3-D shape of these face views.

Angular distance was calculated from the comparison view to the test view through the intersection of the axes (i.e., the front view, see Fig 1). For example, in the ¾ yaw comparison group (where all faces were matched to a 45° right yaw view), a 30° right-yaw test view had an angular distance of 15° from the comparison view, whereas 15° left-yaw, 15° pitch-up and 15° pitch-down test views all had an angular distance of 60° from the comparison view.

Image similarity was measured via both physical calculations and human subjective ratings. The pixel difference measure was calculated as the difference between the mean number of pixels that corresponded to a face in the test and comparison views (averaged across the nine face identities; see S1 Appendix). Human image similarity rating data was obtained for each of the comparison-test face view pairs from a sample of independent participants (see S2 Appendix). Both of these measures were calculated for each of the 21 view conditions for each comparison group.

A measure of perceived 3D face shape for the current stimulus set (from the mid-axis of the head to the tip of the nose) was also obtained from a sample of independent participants. The mean perceived depth differences between the 4 comparison views and 21 test views were then calculated separately for each participant (see S3 Appendix).

Prior to interpreting the results, the data were evaluated for appropriate use in a multiple regression analysis. Inspection of residuals plots indicated that the assumptions of normality, linearity and homoscedasticity of residual were met. Mahalanobis distance and tolerance statistics indicated that multivariate outliers and multicollinearity of predictors were not of concern.

The four variables/predictors (angular distance, pixel difference, perceived image similarity and perceived depth difference) combined accounted for a significant 78.2% of the variance in matching sensitivity (d’), *R*^*2*^ = .782, adjusted *R*^*2*^ = .77, *F*(3,80) = 70.84, *p* < .001. A combined effect of this magnitude, *f*^2^ = .61 can be considered large [35]. As can be seen in Table 2, only image similarity ratings and perceived depth differences were found to be significant predictors of matching performance in the regression model. While pixel difference and angular distance had very little predictive value in this model, there were strong, significant correlations between perceived image similarity and pixel difference and between perceived image similarity and angular distance (r = .68 and r = .74, respectively, both *p* < .001). This suggests that human similarity ratings captured important aspects of both these pixel difference and angular distance measures. Note that a higher image similarity rating indicated a greater perceived difference between the images. Thus, the negative coefficient found for the image similarity variable shows that as the images were rated to be more different, the matching performance decreased. By contrast, a positive coefficient was found for the depth difference variable. This suggests that, after controlling for the other variables/predictors, as the difference in perceived depth between two images increased, so too did the matching performance.

**Table 2:** Regression model. Unstandardised (*B*) and Standardised (β) regression coefficients and squared semi-partial correlations (*sr*^2^) for each predictor in multiple regression analysis predicting matching performance d’.

Variable	B (SE)	β	*sr*^2^
Image similarity	-.47 (.06)**	-.96	.18
Depth difference	.15 (.08)*	.14	.01
Angular distance	.002 (.002)	.09	.003
Pixel difference	-0.000003 (.000004)	-.06	.001

* *p* < .05

** *p* < .001

## General discussion

Unfamiliar face matching across different views was clearly viewpoint dependent. Our findings show that these viewpoint dependent effects varied significantly as a function of the test view axis and test view angle. However, the main finding of this study was the striking differences in face matching performance as a function of which comparison view was being used (i.e., the front, ¾ yaw, ¾ pitch-up or ¾ pitch-down view). That is, some comparison views were better than others for generalisation of face recognition. Overall matching sensitivity was similar for both the front view and ¾ yaw comparison groups and the performance for these two groups was superior to that found for both the ¾ pitch-up and ¾ pitch-down groups.

When views were matched to either a front or a ¾ yaw comparison view, performance (both sensitivity and RT) was significantly better for yaw rotated test views than pitch rotated test views with the pitch-up rotated views showing a steeper decline in performance as a function of test view angle compared to pitch-down rotated views. Indeed, when matching to a ¾ yaw comparison view, performance was consistently high and essentially flat across yaw test views, with the exception of the extra performance advantages found for test views that were identical to, or the mirror of, the comparison view. However, when views were matched to a ¾ pitch-up or a ¾ pitch-down comparison view, there was no overall performance benefit for pitch compared to yaw rotated test views and matching sensitivity did not vary greatly as a function of viewing angle for yaw test views. While there was evidence of a same-view advantage in performance matching to pitch rotated test views for both pitch view comparison groups (most obviously in the ¾ pitch-up comparison view group, see Fig 5, bottom right panel), matching performance appeared to decline rapidly as the angular distance between comparison and test views in the pitch axis increased.

Previous research shows that viewpoint dependent decline in generalising across faces rotated in the pitch axis is greater than for faces rotated in the yaw axis and we have argued that this may be due to an impaired ability to extract or utilise configural or holistic information for faces viewed in pitch rotations [14], [36]. The first aim of this study was to test the generalisability of these findings. Specifically we were interested in whether patterns of viewpoint dependent performance might depend on the comparison view used for the generalisation task. The current results provide very clear evidence that it does. However, while previous research findings from Favelle and colleagues [13], [14] appear to be specific to comparisons made to a front view, the broader conclusion that views of faces rotated in pitch provide poor information for recognition or generalisation still holds. The poorer overall performance observed for the pitch-up and pitch-down (compared to the two other) comparison view groups provides support for this claim. Aside from a same view advantage, pitch comparison views did not generalise well even to other pitch views. We found that: 1) matching pitch comparison views to other pitch test views resulted in a steep viewpoint dependent decline in sensitivity; and 2) this performance was no better than when matching to yaw rotated test views. This shows that previous findings of stronger viewpoint dependent declines in the pitch axis were not unique to matching to the front view, and suggests that performance is likely to be based on differences in the nature of the information available in pitch views of a face compared to yaw. These strong pitch viewpoint costs to face recognition have been found to persist in a recent study by Bülthoff, Mohler and Thornton [37] which allowed participants to dynamically interact with avatars in virtual reality. Even though their interactions with the faces in this study improved overall recognition performance, the representations gained during these more naturalistic experiences still did not prevent strongly viewpoint dependent recognition in the pitch axis.

### Information used to generalise across face views

Performance in a generalisation task may be driven by the quality of the visual information in either of the two views (e.g., internal facial features [38]) or the mechanism used to compare the information in the two views. In this study we tested four potential mechanisms for generalisation across face views: angular distance, physical and human-rated image similarity, and differences in 3D shape information. The results suggested that ratings of image similarity and to a lesser extent differences in perceived face depth were important factors for generalising across views of faces. A multiple regression analysis identified image similarity ratings and perceived depth differences as significant predictors of matching sensitivity. Face matching performance appeared to improve in conditions where: (i) the two face images were rated as being more similar, and (ii) the difference in perceived depth between them was rated to be larger. While the latter positive relationship with perceived depth might seem counterintuitive, it was only marginally significant. One potential explanation of this result is that the front view had one of the lowest estimates of perceived depth but matching with this view was generally very accurate. We note that the conditions with the greatest differences in perceived depth were comparisons between a front view and a yaw rotated view and these were also conditions in which matching performance was high. The accuracy of matching in these cases may depend on access to the face specific information available in a front view rather than depth *per se*.

We note that while the difference in image pixels and the angular distance between views were not identified as significant predictors of matching performance in their own right, these two physical measures were both highly correlated with the human image similarity ratings. This suggests that the higher-order similarity ratings might have captured important aspects of both of these lower-level physical measures. However, these similarity ratings were also likely to have included other physical and other aspects of the image, such as skin tone, appearance/occlusion of features and/or the ease of extracting holistic information. On average, front and yaw comparison views were rated as being more similar to all other test views than either the pitch-up or pitch-down comparison views, following the main effect found for the omnibus analysis. Image similarity ratings for views in both the pitch-down and pitch-up comparison groups, reflect better performance for views within 30° of the comparison view and the equivocal performance across the remaining test views and flat function across yaw test views.

Regardless of how image similarity is measured, the results of this study (gathered over a wide range of viewing conditions) provide support for a solution to the problem of view generalisation in face recognition that involves comparison of 2D image features or interpolation of views rather than mental rotation or alignment to a 3D representation [10], [16]. Even a modified mental rotation account of generalisation that allowed for more efficient rotation in yaw than in pitch cannot explain the current findings of better recognition performance with yaw comparison views and pitch test views than with pitch comparison views and yaw test views (where the rotation between the views would be the same). Findings of differential effects of rotation axis and direction, as well as the lack of explanatory power of perceived depth, are both difficult to reconcile with models using alignment to 3D representations.

### Canonical views of faces

A second aim of this study was to determine whether any of the comparison views tested had canonical status for generalisation of face recognition across views. Previous studies have shown that front and ¾ yaw views generalise well to other yaw rotations [15], [39], however a canonical view should provide superior information and show a generalisation advantage for rotations across orthogonal axes as well as within. In this study the front and ¾ yaw views appear to at least partially meet these criteria, whereas the ¾ pitch views do not. We found that (i) front and ¾ yaw views generalise to some pitch test views just as well as they do to yaw test views, and (ii) there appears to be no three-quarter pitch (up or down) view advantage. Previously, Liu and Chaudhuri [18] have argued that any generalisation advantage or canonical view status of ¾ yaw views may simply be a function of angular distance between views. Contrary to this proposal, our findings indicate that angular distance (beyond what is subsumed by ratings of image similarity) did not account for a significant proportion of variance in performance generalising across views. Furthermore, if angular distance was the primary determinant of a generalisation advantage, then we would see much poorer performance for yaw test views in the ¾ pitch comparison view groups. However, generalisation of face recognition performance in the ¾ pitch comparison view groups is just as good when matching to any yaw test view angle as to other pitch test views.

Assessing the value of intrinsic properties available in various views requires using same-view matching tasks rather than the generalisation (i.e., different view) task used here. And while this is an avenue for future research, we can venture some suggestions as to why ¾ yaw and front views allowed for good generalisation of face recognition across a wide range of test views. Ratings of perceived face depth (S3 Appendix) were largest for yaw views, but much smaller for front views. Thus, the perceived depth of the face, at least as we have measured it, does not fully account for matching performance. Perhaps there are other cues available in yaw views that provide better access to face shape information to aid generalisation. Front and ¾ yaw views contain more visual information (e.g., greater numbers of pixels that correspond to the face) and are rated to be more similar to other views than ¾ pitch views and it may be that the greater amount of visual information in a front and a ¾ yaw view provides more ready access to configural and featural information compared to pitch views [14].

### Implications for photo identity documents

There are clear implications of this research for security systems and photo identity (ID) documents used in those systems. Successful view generalisation of faces is a task central to security and law enforcement work in which novel views of unfamiliar faces (e.g., from photographic evidence, CCTV footage or live viewing) have to be matched to mug shots or other photo ID information. More often than not the photo ID image is a front view and increasingly it is the case that matching has to occur to pitch views (e.g., from CCTV cameras located above head height on street posts or below head height on automatic bank teller machines). Unfamiliar face matching is difficult enough under ideal image conditions of well lit, close distance, front views (see [40] for a review). However, image variability has been shown to improve unfamiliar face matching performance. White, Burton, Jenkins and Kemp [41] have shown that identification of unfamiliar faces (front views) is more accurate with an array of face images to compare (rather than single comparison images) and have argued for the inclusion of multiple images of faces in photo ID documents. A similar effect has been shown for multiple views, with one study showing that learning both a front and profile view of a face lead to better recognition of the intermediate ¾ yaw view than learning either view alone [42]. Our results suggest that a photo ID array that includes both ¾ yaw views and front views of faces would result in the best view generalisation outcomes to novel pitch views as well as yaw.

### Conclusion

The finding that viewpoint effects in face generalisation depend on the axis of rotation is not surprising considering the different demands placed on transformation mechanisms (e.g., occlusions, perturbations of top-bottom or left-right relations). However, not all viewpoint dependent effects show the same pattern and generalisation of face recognition performance is very much reliant on the comparison face view. The current results show that we can generalise well from ¾ yaw and front views to almost all yaw and more test views from an orthogonal (pitch) axis than either of the ¾ pitch comparison views examined here (which do not provide much more than a same view advantage). Previous findings of poorer face recognition with views rotated up or down in the pitch axis are not specific to front view generalisation or to static image comparisons [37]; this poor performance is found even when generalising to other pitch rotated views. Image similarity explains a significant proportion of generalisation performance over and above perceived depth, and together with the differential effects of axis lends support to a view based interpolation approach to the problem of view generalisation for face recognition. With regards to specific views, our results contribute evidence that both the ¾ yaw and front views have intrinsic properties useful for successful generalisation across a broad range of novel views in both yaw and pitch axes and so would be ideally incorporated in photo-identity documents.

## Supporting information

S1 Appendix Raw pixel measure of image similarity. (DOCX)

S2 Appendix Ratings of image similarity. (DOCX)

S3 Appendix Estimate of perceived 3D face shape. (DOCX)

S1 Dataset Sensitivity (d’), Reaction time (ms) and depth rating data. Data is provided for each participant across each test view. Key to the labels for the test views: “zero” refers to the front view, “pd”, “pu”, “ym” and “y” refer to pitch down, pitch up yaw left and yaw right respectively. The number in each test view label refers to the rotation in degrees away from zero.
(XLSX)
