# Supplementary material for: View specific generalisation effects in face recognition: Front and yaw comparison views are better than pitch
Source: PLoS One. 2018 Dec 28;13(12):e0209927. doi: 10.1371/journal.pone.0209927 (PMC6310264; doi:10.1371/journal.pone.0209927)
Supplement: S1 Appendix — (DOCX) [file pone.0209927.s001.docx]

**S1 Appendix. Raw pixel measure of image similarity.**

**Table S1.1.** Mean number of pixels that correspond to the face in each view averaged across the 9 identities (standard deviation in parentheses). The mean value for the front view 0° was 117556 (SD = 5046).

| ***View angle***  ***Axis*** | **15°** | **30°** | **45°*** | **60°** | **75°** | **Mean** |
| --- | --- | --- | --- | --- | --- | --- |
| **Pitch-up** | 109204 (4614) | 94805 (4591) | 77462 (6768) | 54295 (7150) | 35226 (5521) | 74198 (5729) |
| **Pitch-down** | 118261 (3958) | 109359 (6614) | 98457 (6799) | 85224 (7271) | 59711 (10328) | 94202 (6994) |
| **Yaw left** | 113665 (7064) | 116109 (5091) | 111457 (6013) | 105262 (7195) | 98104 (7887) | 108919 (6650) |
| **Yaw right** | 116285 (7047) | 115825 (5272) | 110716 (6458) | 104654 (7535) | 96953 (8605) | 108887 (6983) |

*comparison views
