# Supplementary material for: View specific generalisation effects in face recognition: Front and yaw comparison views are better than pitch
Source: PLoS One. 2018 Dec 28;13(12):e0209927. doi: 10.1371/journal.pone.0209927 (PMC6310264; doi:10.1371/journal.pone.0209927)
Supplement: S2 Appendix — (DOCX) [file pone.0209927.s002.docx]

**S2 Appendix. Ratings of image similarity.**

Similarity rating data was collected from a group of naïve participants (N=18). Participants were undergraduate students at the University of Wollongong who received course credit for participation. Participants rated the similarity of each of the 84 pairs of face views: each of the 4 comparison views (front, 45° right yaw, 45° pitch-up and 45° pitch-down) paired with each of the 21 test views (front view 0° and 15°, 30°, 45°, 60° and 75° views in left and right yaw, pitch-up, pitch-down) used in the experiment. The face pairs were displayed simultaneously on the screen, to the left and right of the centre, and remained on the screen until the participant made a response. The similarity of the two images was rated on a likert scale from 1 (identical images) to 9 (extremely different images). The faces were always the same identity in a pair (each participant saw 3 different identities across the experiment, this was counterbalanced across participant so that all of the 9 identities used in the main experiment were rated) and participants were told this and then instructed make a judgement about how similar the images were. The data are presented in a Table S2.1.

**Table S2.1.** Mean image similarity ratings. A higher number indicates greater difference between images. Note: yr = right yaw, yl = left yaw, pu = pitch-up, pd = pitch-down.

| **Test view** | **Front comparison view** | **Yaw comparison view** | **Pitch-down comparison view** | **Pitch-up comparison view** |
| --- | --- | --- | --- | --- |
| **zero** | 1.09 | 4.19 | 4.76 | 5.70 |
| **pd15** | 3.07 | 4.35 | 3.81 | 5.89 |
| **pd30** | 3.28 | 4.78 | 2.85 | 5.94 |
| **pd45** | 4.76 | 5.43 | 1.17 | 6.37 |
| **pd60** | 5.15 | 5.72 | 2.70 | 6.70 |
| **pd75** | 6.48 | 6.74 | 4.50 | 6.98 |
| **pu15** | 2.74 | 4.44 | 4.86 | 4.57 |
| **pu30** | 3.67 | 4.39 | 5.44 | 3.13 |
| **pu45** | 5.70 | 5.78 | 6.37 | 1.37 |
| **pu60** | 6.67 | 7.11 | 7.09 | 3.74 |
| **pu75** | 7.37 | 7.44 | 7.31 | 5.57 |
| **yl15** | 2.52 | 3.26 | 4.19 | 5.57 |
| **yl30** | 2.89 | 3.30 | 4.56 | 5.19 |
| **yl45** | 4.06 | 2.26 | 4.87 | 5.52 |
| **yl60** | 4.44 | 3.19 | 5.26 | 5.85 |
| **yl75** | 5.01 | 3.19 | 5.52 | 5.20 |
| **yr15** | 2.37 | 2.72 | 4.17 | 5.48 |
| **yr30** | 3.26 | 2.50 | 4.76 | 5.31 |
| **yr45** | 4.19 | 1.24 | 5.43 | 5.78 |
| **yr60** | 4.41 | 2.46 | 5.59 | 5.33 |
| **yr75** | 5.06 | 2.91 | 5.91 | 5.41 |
