# Supplementary material for: View specific generalisation effects in face recognition: Front and yaw comparison views are better than pitch
Source: PLoS One. 2018 Dec 28;13(12):e0209927. doi: 10.1371/journal.pone.0209927 (PMC6310264; doi:10.1371/journal.pone.0209927)
Supplement: S3 Appendix — (DOCX) [file pone.0209927.s003.docx]

**S3 Appendix. Estimate of perceived 3D face shape.**

One of the properties of a three-quarter yaw view that was speculated to drive the three-quarter view advantage was the depth information made available from contour and feature projection (e.g., Hill et al. [4]). To estimate this information, twelve volunteers from the University of Wollongong community (who had not participated in any of the other experiments) were shown 136 face images from the database used to create the stimuli in Experiment 1. Specifically, participants saw 17 views (front view 0° and 15°, 30°, 45°, 60° and 75° views in left yaw, pitch-up, pitch-down plus profile 90° left yaw) of each of 8 face models used in the experiment (one model was omitted by programming error). The stimuli were presented individually at the centre of a computer screen at the same dimensions and resolution as the experiment. For each stimulus, the participant estimated the distance from the mid-axis of the head to the tip of the nose (as an index of perceived 3D face shape). To help describe the distance that was required to be estimated, participants were shown computer generated heads in front and profile views with lines indicating the mid-axis and the protrusion of the nose (see Fig S3.1). Stimuli remained on screen until participants typed the estimate (in cm) in a response box on screen. A 20 cm rule with mm and cm markings was provided for participants to refer to for unit estimation.


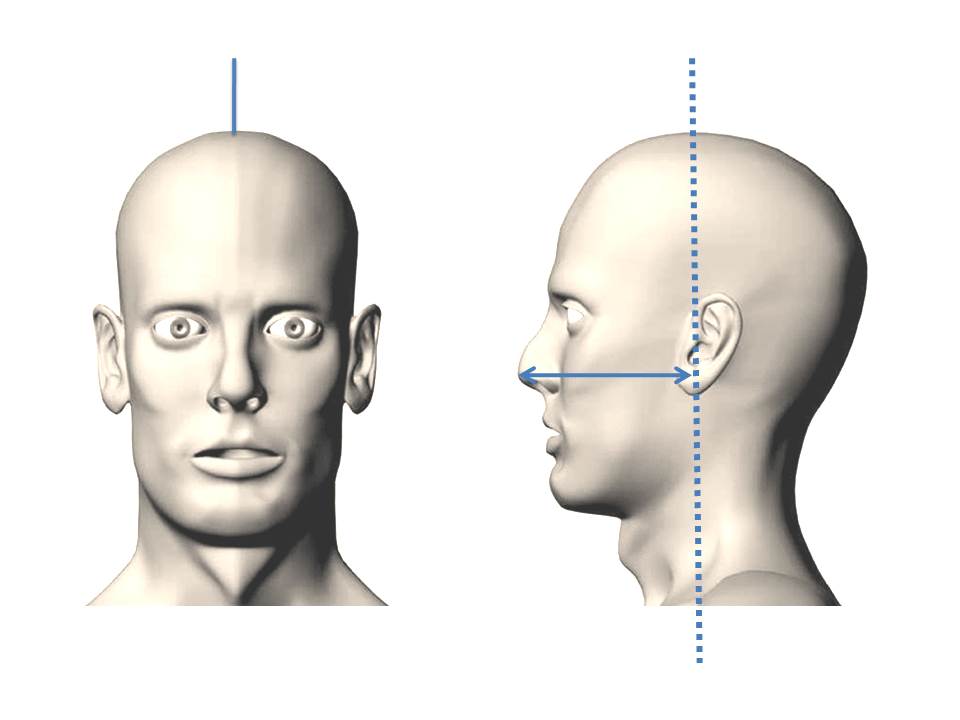


**Fig S3.1.** The explanatory figure provided to participants to help define the distance to be estimated in the task. The arrow on the right head indicates the distance from the mid-axis of the head to the tip of the nose.

The data is presented in Table S3.1. Overall, perceived depth was greatest for the yaw compared to the pitch-up and pitch-down rotated test views. Where perceived 3D depth increases from 15° to 75° in yaw and pitch-down views, the estimate of perceived depth is flat (or slightly decreases) for pitch-up views. The front view has one of the lowest estimates of 3D depth. A one-way repeated measures ANOVA comparing the mean depth estimate of the four comparison views (front, ¾ yaw, ¾ pitch-up and ¾ pitch-down) showed that while there was a significant effect of comparison view, *F*(1.5, 16) = 6.45, *p* = .01, η_p_^2^ = .37, degrees of freedom corrected using Greenhouse-Geisser adjustment, post-hoc pairwise comparisons showed no significant differences between any view pairs (all *p* > .06).

**Table S3.1.** Mean estimation in cm of the distance from the mid-axis of the head to the tip of the nose as a function of axis and view angle (standard deviation in parentheses). The mean value for the front view 0° was 8.56cm (SD = 2.3cm).

| ***View angle***  ***Axis*** | **15°** | **30°** | **45°*** | **60°** | **75°** | **Mean** |
| --- | --- | --- | --- | --- | --- | --- |
| **Pitch-up** | 8.68 (2.2) | 8.88 (1.7) | 8.81 (1.8) | 8.55 (1.9) | 8.03 (2.2) | 8.59 (1.9) |
| **Pitch-down** | 8.60 (2.1) | 9.05 (2.0) | 9.10 (2.0) | 9.44 (1.9) | 9.89 (2.0) | 9.22 (2.0) |
| **Yaw** | 9.30 (1.7) | 9.57 (1.7) | 10.06 (1.5) | 10.68 (1.7) | 11.29 (2.1) | 10.18 (1.8) |

*comparison views
